# LDpop: an interactive online tool to calculate and visualize geographic LD patterns

**DOI:** 10.1186/s12859-020-3340-1

**Published:** 2020-01-10

**Authors:** T. A. Alexander, M. J. Machiela

**Affiliations:** 1 Center for Bioinformatics and Computational Biology, University of Maryland, College Park, MD 20740 USA; 2 Division of Cancer Epidemiology and Genetics, National Cancer Institute, Rockville, MD 20892 USA

**Keywords:** Linkage disequilibrium, Genome-wide association, Geographical visualization, 1000 Genomes Project

## Abstract

**Background:**

Linkage disequilibrium (LD)—the non-random association of alleles at different loci—defines population-specific haplotypes which vary by genomic ancestry. Assessment of allelic frequencies and LD patterns from a variety of ancestral populations enables researchers to better understand population histories as well as improve genetic understanding of diseases in which risk varies by ethnicity.

**Results:**

We created an interactive web module which allows for quick geographic visualization of linkage disequilibrium (LD) patterns between two user-specified germline variants across geographic populations included in the 1000 Genomes Project. Interactive maps and a downloadable, sortable summary table allow researchers to easily compute and compare allele frequencies and LD statistics of dbSNP catalogued variants. The geographic mapping of each SNP’s allele frequencies by population as well as visualization of LD statistics allows the user to easily trace geographic allelic correlation patterns and examine population-specific differences.

**Conclusions:**

LDpop is a free and publicly available cross-platform web tool which can be accessed online at https://ldlink.nci.nih.gov/?tab=ldpop

## Background

Linkage disequilibrium (LD)—the non-random association of alleles at different loci—defines population-specific haplotypes which vary by genomic ancestry [1]. Assessment of allelic frequencies and LD patterns from a variety of ancestral populations enables researchers to better understand population histories as well as improve genetic understanding of diseases in which risk varies by ethnicity. For example, genome-wide association studies (GWAS) identify germline variation associated with disease susceptibility but need to account for ancestry-specific differences in LD patterns when designing the study, analyzing markers and interpreting findings. While population geneticists have developed many datasets (e.g., 1000 Genomes Project, HapMap) [2, 3] and tools (e.g., Geography of Genetic Variants Browser) [4] to investigate differences in allelic frequencies by population group, to date no tool exists to easily explore and visualize LD patterns across 1000 Genomes population groups.

### Implementation

LDpop is an online module designed to allow researchers to query LD patterns of two variants across ancestral populations of interest. LDpop estimates allele frequencies and measures of LD (D′ and R^2^) for each included population. The reference genetic data is from the 1000 Genomes Project Phase 3, which includes sequencing data for 2504 individuals in 26 ancestral populations which are divided into 5 super populations (e.g., African, Ad-Mixed American, East Asian, European, and South Asian) [2]. The 1000G data are available for public download in VCF format (ftp://ftp.1000genomes.ebi.ac.uk/vol1/ftp/release/20130502/).

LDpop is written in Python (2.7.15) and runs on a web-accessible virtual machine with UNIX operating system. The genomic coordinates are retrieved for each query variant from an indexed MongoDb database of dbSNP version 151 and subsequently extracted from the phased 1000 Genomes Project variant call format (VCF) file using Tabix (0.2.5). LDpop uses the Google Maps API to produce the interactive geographic mapping for each population using latitude and longitudinal coordinates for each 1000 Genomes Project ancestral population. The LDpop web-accessible page is programed in HTML5 for cross-browser and cross-platform compatibility and is part of the larger LDlink collection of LD web tools [5, 6]. All code for LDpop is available from out GitHub repository: https://github.com/CBIIT/nci-webtools-dceg-linkage/.

## Results

LDpop takes as input two dbSNP reference SNP numbers (rsIDs), a selection of desired populations from the 1000 Genomes Project, and a choice of which LD statistic (D′ or R^2^) to report for the geographic mapping. LDpop supports queried dbSNP variants which are biallelic including both single nucleotide polymorphism (SNP) and small insertion and deletion (indel) queries. LDpop allows the user to specify any subset of populations from the subpopulations, super populations, and all populations, they are interested in examining for the analysis.

**Fig. 1:**
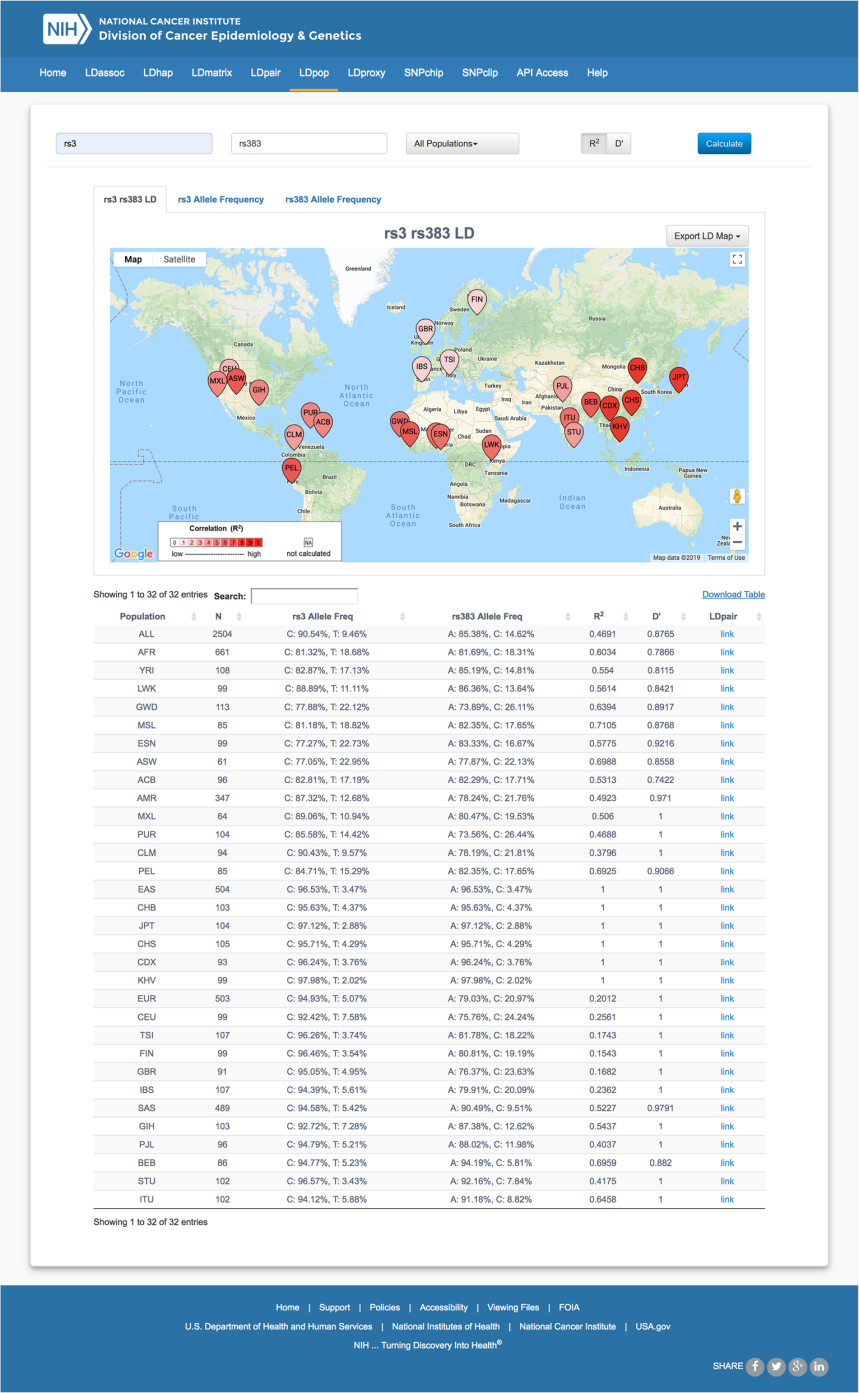
Example of an LDpop interactive map and table. Selected tab displays a map of R^2^ for rs3 and rs383 for all 1000 Genomes Project populations. Numeric data on sample size, allele frequency and LD measures are displayed in the table at the bottom of the screen capture

LDpop produces three geographic maps and one sortable, filterable table as output (Fig. 1). For each queried variant, the allele frequency is calculated for every population selected and the frequency percentage is plotted over the population’s approximate geographic coordinates as a colored pin with deeper blue colors indicating higher allele frequencies. This allows the investigators to easily calculate and visualize changes in allele frequency across ancestral populations for each variant. A LD map is also produced displaying a computed LD statistic (D’ or R^2^) for the two query variants for every population selected. The mapped data point is colored in proportion to the gradient shown in the legend, with darker red signifying a higher degree of LD. All geographic mapping utilizes the Google Maps API for smooth and rapid performance. The interactive summary table at the bottom of the page has a row for each selected 1000 Genomes Project population and displays data in the number of samples in each population, allele frequencies for each variant, and calculated LD values (D′ and R^2^). This table is sortable by column and has a search bar to quickly navigate through it. The table is also downloadable as a text file for local storage and future data integration and analysis.

## Conclusions

LDpop is an online module designed to allow researchers to query LD patterns of two variants across ancestral populations of interest. It is designed to allow users to easily calculate and geographically visualize these LD patterns and changes in allele frequency across ancestral populations. This web tool is freely available and can be accessed at https://ldlink.nci.nih.gov/?tab=ldpop.

## Data Availability

The LDpop web tool is freely available at https://ldlink.nci.nih.gov/?tab=ldpop. The 1000G data are available for public download in VCF format (ftp://ftp.1000genomes.ebi.ac.uk/vol1/ftp/release/20130502/).

## Abbreviations

GWAS: Genome-Wide Association Studies
Indels: Insertion/Deletion
LD: Linkage Disequilibrium
rsIDs: reference SNP numbers
SNP: Single Neucleotide Polymorphism
VCF: Variant call format
